# Share of afghanistan populace in hepatitis B and hepatitis C infection's pool: is it worthwhile?

**DOI:** 10.1186/1743-422X-8-216

**Published:** 2011-05-11

**Authors:** Sanaullah Khan, Sobia Attaullah

**Affiliations:** 1 Molecular Parasitology and Virology Laboratory, Department of Zoology, Kohat University of Science and Technology, Kohat 26000, Khyber Pakhtunkhwa Pakistan; 2 Department of Zoology, Islamia College Peshawar (A Public Sector University), University Campus, Jamrod Road, Peshawar 25120, Khyber Pakhtunkhwa Pakistan

**Keywords:** Hepatitis B virus, Hepatitis C virus, Epidemiology, Risk behaviours, Risk groups, Afghanistan

## Abstract

There is a notable dearth of data about Hepatitis B Virus (HBV) and Hepatitis C Virus(HCV) prevalence in Afghanistan. Awareness program and research capacity in the field of hepatitis are very limited in Afghanistan. Number of vulnerabilities and patterns of risk behaviors signal the need to take action now.

Thirty one studies dating from October 2003 to 2011 were included, consisting the data of 1,32,981 individuals for HBV and 1,32,500 individuals for HCV. Percentage prevalence was 1.9% for HBV and 1.1% for HCV in all available Afghanistan population. Most at risk population to hepatitis include injecting drug users who share needles and female sex workers, while truck drivers, prisoners and homosexual men needs attention, as their statistical figure are missing. Data suggests that high incidence of intravenous drug use, sexual activities, unsafe blood transfusion procedures and mobility are major risk factors for hepatitis transmission.

This review is based on analysis of the limited available data in Afghanistan. Although there are many underlying vulnerability factors, it appears that Afghanistan remains at an early epidemic phase. Further research is required to determine the seroprevalence and prevalent genotype(s) of HBV and HCV in all provinces in Afghanistan. This article provides some key insights into the potential and likely future transmission dynamics of Hepatitis which will serve as a guide in the identification of priority areas in term of high risk groups and risk behaviours in the country and will assist to develop urgent strategic plans to combat the future burden of Hepatitis in Afghanistan.

## Introduction

Both HBV and HCV are major cause of dreadful liver diseases including acute hepatitis, chronic liver diseases and hepatocellular carcinoma [1]. It is estimated that 30% of the global population (about 2 billion persons) have serologic evidence of HBV infection while over 350 million people are carriers of chronic HBV worldwide [2]. Approximately 3% of the world's population (approximately 170 million people) have chronic hepatitis C virus (HCV) infection and 3-4 million people are newly infected annually [3].

Afghanistan is the world's 41^st ^largest country of about 28,150 million inhabitants [4] and fertility rate of 6.5 children per woman [5]. It is among the least developed countries in the world, with hampering factors like porous borders, generally low levels of education and literacy rate, and limited health and social infrastructure. Over the past few decades, Afghanistan has endured widespread armed conflict resulting in extensive internal and external displacement of people, and social and economic upheaval [4]. Hundreds of thousands of people are internally displaced by conflict and natural disasters, residing in camps and cities across Afghanistan. In addition, external displacement is also significant, particularly refugees to neighbouring countries including Iran and Pakistan [6], which harbor higher infected population of HBV and HCV [7,8]. Higher prevalence of hepatitis viruses has been reported among Afghan refugee populations outside of Afghanistan [9] may raise the concern of elevated prevalence of hepatitis within the country.

Due to more than 20 years of political and wartime unrest, the information about epidemiology of HBV and HCV in Afghanistan has become completely out of date. Insofar as it is possible through the use of existing limited data, this article addresses the epidemiology of HBV, HCV and genotypes and identification of risk factors in Afghanistan. The main objective of this article is to inform policy makers and program managers about trends in HBV and HCV rates and risk behaviours over time across different regions and population groups which are very useful for developing proper preventive guidelines and educational programs.

### Literature search

All available data of interest till the writing of this review article from 2003 to March 2011 were searched for in PubMed database of the National Library of Medicine, National Institutes of Health (USA), using two search strategies: (Hepatitis and Afghanistan) and (HBV or HCV or Blood Borne and Afghanistan). Inclusion criteria entailed the studies demonstrating the prevalence, genotypes and risk factors of HBV and HCV in the Afghanistan population. To provide a simple review of the current situation, thirty one different articles/abstracts/reports were obtained from different sources, including national and international reports and scientific publications. Data from these articles regarding study time period, region (city and province), study population, total sample size, and percentages and numbers of HBV and HCV positive cases were extracted. All literature identified by search were read and categorized into 2 groups: A) Prevalence of HBV and HCV in various population B) HBV and HCV genotyping and C) High risk groups and risk behaviour.

## A. Prevalence of HBV and HCV in various populations

Few studies have addressed the prevalence of HBV and HCV from different areas of the country in the various population of Afghanistan. Identification and comparison of data in this article helps the decision makers reach a better understanding of the current picture among subpopulation throughout the country.

### i. Injecting Drug Users (IDUs)

Only two studies dealing with the frequency of HBV among IDUs were found, covering 1,087 IDUs from Kabul, Hirat, Jalalabad, and Mazar-i-Sharif cities from period of June 2005 to 2010. HBV serofrequency in these individuals ranged from 5.8-6.5%, with an overall prevalence of 6.15% [10,11].

Three studies from period of June 2005 to 2010 covering 1,696 IDUs from Kabul, Hirat, Jalalabad, and Mazar-i-Sharif cities showed a range of 36.0-36.6% HCV serofrequency. The mean frequency was 36.4% and 1.5% were found co-infected with HIV and HCV [10-12]. The data on the disease burden in this large high-risk population are limited while growing number of IDUs in Afghanistan places the country at great risk for epidemics of hepatitis in future.

### ii. Intrapartum patients

The study in 2006-2007 was among the first and last till the writing of this article to assess prevalence of hepatitis B surface antigen (HBsAg) and HCV in 4,452 obstetric populations admitted at three Kabul hospitals for obstetric indications. A survey found 1.53% positive cases of HBsAg and 0.3% cases of HCV and none was positive for both. It was concluded that prevalence rate was lower, probably because this study comprised only those women who were able to access care, likely belonged to higher socioeconomic status. Because hepatitis has been associated with lower socioeconomic status, the prevalence rate reported in this study may underestimate the true prevalence among reproductive-aged women in Kabul. Prevalence of the infection among the antenatal population may be a reliable indicator of general population prevalence. The intrapartum period of the antenatal population may be the only clinical access point in a variety of limited resource settings thus their routine surveillance should be considered important [8].

### iii. Sex workers

In only one study the HBV and HCV serofrequency in 520 Female Sex Workers (FSWs) from Jalalabad, Kabul, and Mazar-i-Sharif was investigated. The prevalence rate of HCV was 1.92%, and HBV was 6.54% in this subgroup [13]. However, no study in male sex workers was identified.

### iv. Blood Donor and General Population

Blood donor prevalence data is one of the published sources of population-based prevalence data [8]. 1,25,832 blood donors were tested by Central Blood Bank Kabul and its provincial branches during the years 1989-2005 and reported 2,221 (1.76%) were positive for HBsAg and 795 (0.63%) for HCV (Table 1). Central Blood Bank reported HBsAg in 3.9% and anti-HCV in 1.9% Afghanistan blood donors from March to December 2006. Blood donor prevalence data is one of the published sources of population-based prevalence data [8,14]. More recent data about the blood donors are not available. There is no information on the prevalence rates of Hepatitis amongst general population due to the absence of sentinel surveillance sites and case reporting systems in Afghanistan. Studies of Hepatitis prevalence conducted in blood donor populations may not truly represent the prevalence in general population. Prevalence in blood donors may be an underestimate the prevalence if potential donors with a high-risk profile (like history of jaundice, injection drug use, multiple sexual partners, etc.) are screened out by questionnaires. Conversely, prevalence may be an overestimate the general population prevalence if professional blood donors were included, who are often IDUs and selling blood for money [15].

**Table 1:** Positive serologic tests done in the central bank and its Provincial branches during the years 1989-2005

Years	Tested Areas	Total tested	HCV	HBS
1996	Central Blood Bank	5309	6	71

1997	Central Blood Bank	3942	10	50

1998	Central Blood Bank	4221	16	97

1999	Central Blood Bank	7964	13	137

2000	Central Blood Bank	6844	1	280

2001	Central Blood Bank	6691	0	114

2002	Central Blood Bank	11586	4	248

2002	Parwan	581	0	5

2002	Mazar-e-Sharif	964	0	32

2003	Central Blood Bank	10674	54	173

2003	Nangarbar	3629	57	194

2003	Mazar-e-Sharif	4714	0	12

2004	Central Blood Bank	10514	80	190

2004	Nangarhar	3525	90	238

2004	Baghlan	232	0	9

2004	Mazar-e-Sharif	2147	1	17

2004	Heart	2890	3	6

2004	Jozjan	1264	1	77

2004	Logar	888	5	45

2004	Badakshan	256	1	18

2005	Central Blood Bank	7684	206	107

2005	Nangarhan	2145	148	49

2005	Heart	2185	23	4

2005	Mazar-e-Sharif	1190	7	1

2005	Juzjan	584	42	2

2005	Parwan	168	8	0

2005	Kunar	11	1	0

2005	Qandahar	1867	2	7

2005	Kunduz	826	16	38

**Total**	**Blood samples tested for HBV and HCV till 2005**	**125,832**	**795**	**2221**

### v. Health Care Workers (HCWs)

Only one published data was found addressing the HBV infection in 113 HCWs from obstetrics-gynecology and pediatric staff, Women's Hospital, Kabul in 2005. The survey reported that 23% HCWs had evidence of previous exposure to HBV. This survey was unable to test HCWs for HCV, but concerned that it may also be present as comorbid conditions since HCV share transmission patterns with HBV. Only two studies pertained to HBV vaccination status. In 2004 only 8% reported a history of previous hepatitis B immunization [16] and recently 27.9% HCWs in 10 national public hospitals in Kabul had not vaccinated against HBV [17].

### vi. Afghan refugees

Refugees may differ from the general population which might affect their risk of HBV infection. For example, the circumstances those lead to refugee status including fleeing from violence and their socioeconomic status as they have the resources and opportunity to leave their country of origin. The prevalence rate of HBsAg was 4.1% and 5% among Afghan refugees in the United States between 1979-1991 and 2007-2008, respectively [18] and 60.8% (45/74) among Afghan refugees in Dalaki, Iran [19]. In 2003, out of 903 Afghan refugees in Balochistan, Pakistan, 8.3% (95% CI: 6.6-10.3) were found positive for HBsAg.

Most of the Afghan refugees in USA were of higher socio-economic class living and one of the probable reasons of high seroprevalence of HBsAg among refugees in Baluchistan was due to increased use of unsafe injection practices and higher socio-economic status of most of refugees in USA probably with better healthcare behavior [9]. Their higher status could potentially lessen the likelihood of HBV infection, because prevalence has been shown to be inversely related to socioeconomic status [18].

## B. HBV and HCV genotypes

Hepatitis B is present everywhere in Asia and is endemic in Afghanistan [20]. While the most prevalent HBV genotype reported in southeastern Asia is D [21]. Only one study based on small sample sizes (N = 12) revealed that HBV genotype D, sub-genotype D1 (98% bootstrap value), and serotype ayw2 were found among HBV infected patients in Afghanistan [22]. No study is available on geographic variation in the prevalence of various HBV and HCV genotypes and their routes of transmission from Afghanistan.

## C. High risk groups and risk behaviour

Several issues are related to estimate risk for specific causes of Hepatitis in world but still in Afghanistan the evidence for its causes are scanty. An effort is made to better understand and identify the risk factors which are important for setting up control measures in the country.

### i. Socio-Economic aspects of country

Afghanistan is the second least developed country in the world [23] and falls at the bottom of the 177 countries according to the Human Development Index [6]. Unemployment, political instability, poverty and low literacy are linked to initiation of injecting and sex trade [24]. In Kabul unemployment were found to be independent risk factors for HCV among younger injectors [25] and 90% commercial sex workers (CSWs) gave poverty as the reason of their involvement in sex work [6].

### ii. Drug abuse and needle or syringe sharing

Throughout the world about 8-16 million HBV and 2.3-4.7 million HCV infections may result from unsafe injections annually [26]. About 50 - 100% of IDUs are anti-HCV positive globally, thus constitute one of the potential reservoirs in community [27]. 88% of the world's opium supply in 2005 was recorded from Afghanistan, the main center of opium production [10]. Drug dependency continues to increase equally across rural and urban areas due to widespread and easy access to cheap drugs and limited access to drug treatment, combined with three decades of war-related trauma have resulted in problem drug-use among almost one million Afghans, roughly 8% of the population. It was stated that approximately 2 out of every 1,000 urban adult men were identifiable IDUs. Overall, 1,465 active IDUs were mapped in the Kabul, Jalalabad and Mazar-i-Sharif and expected significant increase in the number of drug users [24].

Increase in injection drug use and needle sharing pave the way for the spread of hepatitis infection [10]. 87% of IDUs had shared needle and syringe with other injectors, 60% used needle and syringe that had been already used by 2-5 people [28]. It is highlighted the need of drug counseling and educational efforts to older drug users who have not yet initiated injecting and to young IDUs to avert infection and reduce risky drug use behaviors [25].

### iii. Sexual activities and unsafe sex

Sexual transmission of infection is more frequent among high-risk sexual behaviors groups such as multiple sexual partners [27], working as a prostitute, homosexuals and non-protected sexual contact [6,24]. FSWs in Afghanistan are highly vulnerable, diverse and generally have much larger sexual networks. There were about 1.9 FSWs per 1,000 women only in Kabul, Mazār-i-Sharif and Jalalabad cities [24], 84% of FSWs had 1-2 clients/day [4] and with 15 clients/month, 200 FSWs will have 3,000 sexual encounters/month, and more than 35,000/year [24] with stark predictions that the number will be on rise as women and girls resort to selling themselves to escape poverty [4]. Some truck drivers do engage in homosexual sexual relation with their generally young conductors (assistants) [6]. Male IDUs also reported having both commercial sex and sex with other men [24]. CSWs are sexual partners of IDU and at time IDU themselves [6]. Behavior of multiple sexual partners seems to be very important in Afghani IDUs and truck drivers, which may spend long periods away from home and family and play as a source of infection for the whole community [6].

Limited use of condom was reported in the country [29]. A survey in 2005 reported less than 1% use of condoms among CSWs [6], in 2009 only 17-32% of IDUs used condom in their last sexual encounter (in last 6 months) and in 2009 only 51% of truck drivers who buying sex used condoms [4]. Globally street children have been considered a high risk group for acquiring and transmitting infectious diseases like HIV because their lifestyle and daily struggle for food, shelter and safety may lead them to provide sexual favours to obtain the necessities of life or to drug taking, to dull the impact of hardness of life [6] and still no data exist regarding to them.

### iv. Occupational exposure in Health care settings

HCWs are dealing with contaminated blood, blood-related products and exposure prone procedures. Occupation injuries in healthcare settings have been well described in the literature as a major mode of transmission of HBV and HCV in developing countries [2]. HCWs in Afghanistan are particularly disadvantaged due to decades of neglect of their personal health care and widespread inadequate infection control practices in country, as well as no precise national data on the number of needle sticks. WHO modeled was applied to world census which showed that about 66,000 HBV and 16,000 HCV infections may result from occupational sharps in HCWs each year. For the country grouping in which Afghanistan is placed, these estimates are 6,800 for HBV and 3,200 for HCV. These figures were shown to be substantially lower in regions where strict efforts have been made to reduce occupational exposures [16]. In a survey, 72.6% hospital staff reported sharps injury in the preceding 12 months, mainly from hollow-bore needle injuries (46.3%), particularly during re-capping (24.5%) [17]. The medical histories and current symptoms were all self-reported and, in some cases, may represent under-reporting [16].

### v. Unsafe health care practices

HCV has been considered a potential health hazard for military troops and veteran, because reliable screening of blood products became possible in 1993 in Afghanistan and still remains little developed [30]. Large parts of the population do not have access to screening therefore blood transfusions had undertaken without any screening. About 60,000 transfusions are usually documented annually with 12000-16,000 in Kabul alone, of which not more than 30% of blood donations were screened [6]. Consequently the poor state of blood transfusion facilities throughout the country is of primary concern in the control of the spread of Hepatitis and AIDS [6]. No systematic data on the prevalence of transmitted infections are available due to absence of surveillance in Afghanistan.

### vi. Mobile groups (refugees, internally displaced persons (IDPs), truck drivers and migrant workers)

Rapid population mobility and migration is reducing the differences in infectious disease epidemiology between different regions of the world [31]. In Afghanistan certain mobile populations particularly refugees and IDPs are at higher risk of infection for various reasons, including exposure to sexual abuse, violence, and lack of access to information and education [6]. It is evidence that high proportions of IDUs are mobile across international borders to Iran and Pakistan where hepatitis is epidemics in IDU population. It could hasten the initiation of epidemics in Afghanistan [32]. However, there are no systemic data available on prevalence of hepatitis amongst Afghan mobile population.

Afghans comprise the second largest number of refugees and IDPs in the world. Currently, an estimated 440,000 people were displaced by conflict and natural disasters across Afghanistan [24]. Because of returning refugees, the population of Kabul has increased to ≈3 million since 2001, and estimated 470 IDUs in 2003 [8]. High influx of returning refugees is also said to be a factor of bringing the virus with them [9].

Most of IDUs Afghan had traveled outside Afghanistan in the previous 10 years [8]. Almost 69% of IDUs in Mazar-i-Sharif and 80% in Jalalabad had lived outside Afghanistan. This is an area of critical concern that neighbour countries have reported outbreaks of hepatitis among IDUs [24], may place other communities at risk upon their return [8].

About 1,000,000 population leave Afghanistan for working purposes annually to neighboring countries consisting significant burden of blood born infection [6]. About 60,000 domestic truck drivers and 2,000 are international truckers in Afghanistan [4]. International experience suggested that truck drivers do engage the services of CSWs and generally use drugs to overcome fatigue associated with the long hours of long distance driving. Sex industry trends to be most flourished in mover's destination location [6,31]. The evidence showed that population mobility has an independent risk factor for spread of infectious diseases, because they can transfer the disease to the other places where they move to. Afghan truck drivers is high risky group for acquiring and transmitting HIV as they travel along such routes and into neighbor countries, all of which have large or developing sexually transmitting infection problems [6]. Despite of high-risk behavior, no prevalence data is reported for HBV and HCV among them Workers from neighbouring countries also come into Afghanistan to work but no data available on risk behaviours and prevalence of hepatitis amongst Afghan migrant workers [6].

### vii. Prisoners

Imprisonment is the strongest predictors of hepatitis in some areas of Asia [32]. Prisons are extremely high-risk environments for infectious disease because of overcrowding, poor nutrition, limited access to health care, continued drug use and unsafe injecting practices, unprotected sex and tattooing. In 2007, 10,590 prisoners and detainees were reported in 35 prisons of Afghanistan [4] and 126 female prisoners were in Kabul prison, of which 60-70 were arrested due to involvement in sex work [24]. In most cases prisons are linked to the sharing of injecting equipment and to unprotected sexual encounters as access to clean needles and condoms are limited there [6]. One third of Afghan IDUs had been reported drug injection in prison [8,11,29].

### viii. Knowledge

The basic knowledge of universal precaution was insufficient across all investigated hospitals and cadres in Kabul [17,33]. 72.1% of obstetric patients and 91.1% of HCWs were aware of HBV while correct transmission knowledge of HBV was found in 1.9% for patients and 33.9% for HCWs in Afghanistan [33]. In a survey in Kabul, obstetric care providers underutilize testing of sexually transmitted infections for antenatal patients in Afghanistan, due to presumed rarity of infections and due to logistical and cultural barriers [34].

## Discussion

There has been paucity of community based epidemiologic work in Afghanistan. Very few studies have addressed the magnitude of HBV and HCV in the high-risk population including IDUs, FSWs, blood donors and Afghan refugees living in the various parts of the world. There are many areas where research cannot be carried out at all, including general population, household contacts of HBV and HCV infected patients, multi-transfused population of patients with thalassemia or hemophilia, patients undergoing chronic dialysis. Without a deeper engagement with all these population, it is difficult to offer extensive programmatic guidance for their safe guard. Significant work is required to conduct a comprehensive mapping to identify the prevalence rate in Afghanistan. Due to lack of data from large parts of county, exact figures are missing, thus stringent efforts are required for national surveillance to assess the exact situation. The country seems to be at an early phase of HBV and HCV prevalence, but there are a number of underlying vulnerability factors that could lead to the conditions for epidemic, including war instability, huge population displaced internally and externally, drug trafficking, extremely low socio-political and economic status, a fledgling health care system, and a low level of knowledge and awareness about hepatitis.

Over three decades of protracted armed conflicts have caused massive increases in poverty, insecurity and the breakdown of social structures in the country [16]. It facilitated a dramatic increase of illegal production and marketing of opiates. Although non-injection use of opium (smoking, vaporization, or oral ingestion) is traditional in Afghanistan, as with analgesic and relaxing properties considering it a potential source of relief for all kinds of stress. But the intensification of the war on drugs through the reduction in heroin availability may push more drug users towards injecting [8]. In study in 2003, 50% of participants had started using heroin in either Pakistan or Iran [15,27].

Among the various routes of transmission, shared needles by IDUs is considered the key driver in Afghanistan [8]. Almost 80% of IDUs are known to be mobile, that they had changed residence at least once and 70% of IDUs had shared needles [4]. Furthermore, HCV infected IDUs were more likely to have had injections from non-medical providers, which has been linked to Pakistan [8]. Furthermore, this is a disturbing finding as IDUs often trade sex for drugs. Individuals who fall into both categories are therefore particularly vulnerable to HBV and HCV and are perhaps doubly stigmatized [28]. The immigration of populations from neighboring countries of high endemic of the infection seems to contribute significantly to the rapid modification of epidemiological data. The high-risk behavior of injection drug use in refugees may place communities at risk upon their return [10]. IDUs are numerous in society, though they have been inadequately studied and limited data suggest the likelihood that the prevalence of hepatitis is very high in this community.

The main problems in country are high rate of illiteracy, unawareness about sexually transmitted diseases and low use of condoms among the IDUs and sex workers that could lead to spill-over to other high risk groups and from there to the general population [4]. Moreover, extremely poor health structure regarding blood transfusion and universal precautions would seem favorable to a rapid spread of this disease if there is not urgent intervention. Providing occupational safety and health training to HCWs is one avenue to improve security [16]. It leads officials to warn of the urgent need of the effective programs for early interventions to prevent the spread of hepatitis in Afghanistan. There is a need to focus on behavioral sentinel surveillance activity and inventory program for the improvement of HBV and HCV awareness and prevention in country as a matter of urgency.

## Conclusion

Currently no systematic country wide data is available about the actual prevalence rate of HBV and HCV and its genotypes. In order to delineate the risk groups of HBV and HCV it is necessary to conduct epidemiological studies that depict an accurate frequency of the disease in the subpopulation. A high proportion of IDU in Afghanistan are mobile across international borders to Iran and Pakistan suggests that linkages to hepatitis epidemics in IDU in those countries could hasten the initiation of epidemics in Afghanistan. There is the risk that IDU may spread viral hepatitis from to the general public, and then propagated through unprotected sex alongside needle-sharing and unscreened blood transfusion. This situation needs to be addressed immediately, as a policy and political prime issue at the country and all community levels. Drug addicts and unsafe sex activities should be of prime importance as the targets of disease prevention and control programs and are considered as mobile source of disease transmission.

## Competing interests

The authors declare that they have no competing interests.

## Authors' contributions

SK and SA designed, searched literature and gave a critical view of manuscript writing. Both the authors' read and approved the final manuscript.

## References

[B1] Krasteva A Panov VI Garova M Velikova R Kisselova A Krastev Z Hepatitis B and C in Dentistry Journal of IMAB - Annual Proceeding 2008 14 book 2 38 40 (Scientific Papers)

[B2] Singhal V Bora D Singh S Hepatitis B in health care workers: Indian scenario Journal of Laboratory Physians 2009 1 2 41 48 10.4103/0974-2727.59697 PMC3167966 21938248

[B3] Mukhopadhya A Hepatitis C in India J Biosc 2008 33 465 473 10.1007/s12038-008-0065-0

[B4] United Nations General Assembly Special Session on HIV/AIDS, Country Progress Report (Reporting Period January 2008 to December 2009) 2010 Islamic Republic of Afghanistan

[B5] UNICEF Report, Statistics of Afghanistan 2010

[B6] Afghanistan National Strategic Framework for HIV/AIDS National HIV/AIDS and STI Control Programme (2006-2010), Working Draft 05/07/08, National AIDS Control Program 2008 Ministry of Public Health, Islamic Republic of Afghanistan

[B7] Zafar T Brahmbhatt H Imam G Hassan S Strathdee S Epidemiology and Social Science HIV Knowledge and Risk Behaviors among Pakistani and Afghani Drug Users in Quetta, Pakistan Journal of Acquired Immune Deficiency Syndromes 2003 32 4 394 398 10.1097/00126334-200304010-00008 12640197

[B8] Todd CS Ahmadzai M Atiqzai F Miller S Smith JM Ghazanfar SA Strathdee SA Seroprevalence and correlates of HIV, syphilis, and hepatitis B and C virus among intrapartum patients in Kabul, Afghanistan BMC Infect Dis 2008 17 8 119 10.1186/1471-2334-8-119 PMC2557011 18798996

[B9] Quddus A Luby SP Jamal Z Jafard T Prevalence of hepatitis B among Afghan refugees living in Balochistan, Pakistan International Journal of Infectious Diseases 2006 10 3 242 247 10.1016/j.ijid.2005.04.007 16448838

[B10] Todd CS Abed AMS Strathdee SA Scott PT Botros BA Safi N Earhart KC Prevalence of HIV, hepatitis C, hepatitis B, and associated risk behaviors among injection drug users in Kabul, Afghanistan Emerg Infect Dis 2007 13 9 1327 31 18252103 10.3201/eid1309.070036 PMC2857281

[B11] Nasir A Todd CS Stanekzai MR Bautista CT Botros BA Scott PT Kim JH Strathdee SA Tjaden J Prevalence of HIV, hepatitis B and hepatitis C and associated risk behaviours amongst injecting drug users in three Afghan cities Int J Drug Policy 2011 22 2 145 52 10.1016/j.drugpo.2010.10.006 21146392 PMC7243866

[B12] Nasir A Todd CS Stanekzai MR Bautista CT Botros BA Scott PT Kim JH Strathdee SA Tjaden J Implications of hepatitis C viremia vs. antibody alone on transmission among male injecting drug users in three Afghan cities Int J Infect Dis 2011 15 3 e201 5 10.1016/j.ijid.2010.11.006 21190883 PMC7153690

[B13] Todd CS Nasir A Stanekzai MR Bautista CT Botros BA Scott PT Strathdee SA Tjaden J HIV, hepatitis B, and hepatitis C prevalence and associated risk behaviors among female sex workers in three Afghan cities AIDS 2010 24 S69 S75 10.1097/01.aids.0000386736.25296.8d 20610952 PMC3650731

[B14] Central Blood Bank Report of testing of Blood donors from March-December, 2006 Ministry of Public Health Afghanistan

[B15] Ali SA Donahue RMJ Qureshi H Vermund SH Hepatitis B and hepatitis C in Pakistan: prevalence and risk factors Int J Infect Dis 2009 13 1 9 19 10.1016/j.ijid.2008.06.019 18835208 PMC2651958

[B16] Kitt MM Khalid G Rahimi S McCarthy BJ An Occupational Health Services Initiative at a Women's Hospital in Kabul Afghanistan Public Health Rep 2006 121 6 650 657 10.1177/003335490612100604 PMC1781906 17278399

[B17] Salehi AS Garner P Occupational injury history and universal precautions awareness: a survey in Kabul hospital staff BMC Infect Dis 2010 10 19 10.1186/1471-2334-10-19 20113517 PMC2835705

[B18] Rein DB Lesesne SB O'Fallon A Weinbaum CM Prevalence of Hepatitis B Surface Antigen Among Refugees Entering the United States Between 2006 and 2008 Hepatology 2009 51 1 4 10.1016/j.jhep.2009.04.007 19902482

[B19] Pourkarim MR Zandi K Davani NA Pourkarim HR Amini-Bavil-Olyaee S An aberrant high prevalence of hepatitis B infection among Afghans residing in one of the Bushehr refugee camps (Dalaki camp) in the southwest of Iran Int j Infect Dis 2008 12 1 101 2 10.1016/j.ijid.2007.03.008 17540600

[B20] Wright TL Introduction to Chronic Hepatitis B Infection Am J Gastroenterol 101 S1 S6 10.1111/j.1572-0241.2006.00469.x 16448446

[B21] Baig S Siddiqui A Chakravarty R Moatter T Hepatitis B virus subgenotypes D1 and D3 are prevalent in Pakistan BMC Res Notes 2009 4 2 1 10.1186/1756-0500-2-1 PMC2636821 19121226

[B22] Amini-Bavil-Olyaee S Alavian SM Adeli A Sarrami-Forooshani R Sabahi F Hepatitis B virus genotyping, core promoter and precore/core mutations among afghan patients infected with hepatitis B: a preliminary report Journal of Medical Virology 2006 78 3 358 364 10.1002/jmv.20547 16419114

[B23] Human Development Report 2009 Foreword Overcoming barriers: Human mobility and development United Nations Development Programm (UNDP 2009) Retrieved 14/11/2010

[B24] South Asia Human Development Sector, Mapping and Situation Assessment of Key Populations at High Risk of HIV in Three Cities of Afghanistan. Report No. 23 SAR AIDS (South Asia Regional AIDS), Human Development Sector South Asia Region

[B25] Bautista CA Todd CS Abed AMS Botros BA Strathdee SA Earhart KC Safi N Scott PT Effects of duration of injection drug use and age at first injection on HCV among IDU in Kabul Afghanistan Journal of Public Health 2010 32 3 336 341 10.1093/pubmed/fdq020 20421237

[B26] Alam S Ahmad N Khan M Mustafa G Al Mamun A Mashud G Seroprevalence of Hepatitis C Virus Infection among Health Care Workers J Bangladesh College of Physicians and Surgeons 2007 25 3 126 129

[B27] Alavian SM Fallahian F Lankaran KB Epidemiology of Hepatitis C in Iran and the World Shiraz E-Medical Journal 2007 16 4 403 406 18193122

[B28] Afghanistan Female Prisoners and their Social Reintegration UNITED NATIONS New York, UNITED NATIONS OFFICE ON DRUGS AND CRIME Vienna Project: AFG/S47--Developing Post-Release Opportunities or Women and Girl Prisoners 2007

[B29] Todd CS Nasir A Stanekzai MR Abed AMS Strathdee SA Bautista CT Scott PT Botros BA Tjaden J Prevalence and correlates of syphilis and condom use among male injection drug users in four Afghan cities Sexually Transmitted Diseases 2010 37 11 719 25 10.1097/OLQ.0b013e3181e2c76a 20585276 PMC7153692

[B30] Wallace MR Hale BR Utz GC Olson PK Earhart KC Thornton SA Hyams KC Endemic Infectious Diseases of Afghanistan Oxford Journals Medicine Clinical Infectious Diseases 2002 34 5 S171 S207 10.1086/340704 12019465

[B31] Hugo G Indonesia internal and international population mobility: implication for the spread of HIV/AIDS 2001 HUMAN DEVELOPENT REPORT 2009 South East Asia HIV and Development Office ILO, Indonesia, UNAIDS Indonesia

[B32] Alavi M Behdad F Seroprevalence Study of Hepatitis C and Hepatitis B Virus among Hospitalized Intravenous Drug Users in Ahvaz, Iran (2002-2006) Hepatitis Monthly 2010 10 2 101 104 PMC3270351 22312381

[B33] Todd CS Ahmadzai M Atiqzai F Smith JM Miller S Azfar P Siddiqui H Ghazanfar SA Strathdee SA Prevalence and correlates of HIV, syphilis, and hepatitis knowledge among intrapartum patients and health care providers in Kabul, Afghanistan AIDS Care 2009 21 1 109 17 10.1080/09540120802068779 19085227 PMC7183539

[B34] Todd CS Ahmadzai M Smith JM Siddiqui H Ghazanfar SA Strathdee SA Attitudes and practices of obstetric care providers in Kabul, Afghanistan regarding antenatal testing for sexually transmitted infection J Obstet Gynecol Neonatal Nurs 2008 37 5 607 15 10.1111/j.1552-6909.2008.00283.x 18811781 PMC7183538

